# The C313Y Piedmontese mutation decreases myostatin covalent dimerisation and stability

**DOI:** 10.1186/1756-0500-4-442

**Published:** 2011-10-24

**Authors:** Carlene S Starck, Andrew J Sutherland-Smith

**Affiliations:** 1 Institute of Molecular BioSciences, Massey University, Private Bag 11-222, Palmerston North 4442, New Zealand; 2 The Hospital for Sick Children, 555 University Ave, Toronto ON, M5G 1X8, Canada

## Abstract

**Background:**

Myostatin is a key negative regulator of muscle growth and development, whose activity has important implications for the treatment of muscle wastage disorders. Piedmontese cattle display a double-muscled phenotype associated with the expression of C313Y mutant myostatin. *In vivo*, C313Y myostatin is proteolytically processed, exported and circulated extracellularly but fails to correctly regulate muscle growth. The C313Y mutation removes the C313-containing disulphide bond, an integral part of the characteristic TGF-β cystine-knot structural motif.

**Results:**

Here we present *in vitro *analysis of the structure and stability of the C313Y myostatin protein that reveals significantly decreased covalent dimerisation for C313Y myostatin accompanied by a loss of structural stability compared to wild type. The C313Y myostatin growth factor, processed from full length precursor protein, fails to inhibit C2C12 myoblast proliferation in contrast to wild type myostatin. Although structural modeling shows the substitution of tyrosine causes structural perturbation, biochemical analysis of additional disulphide mutants, C313A and C374A, indicates that an intact cystine-knot motif is a major determinant in myostatin growth factor stability and covalent dimerisation.

**Conclusions:**

This research shows that the cystine-knot structure is important for myostatin dimerisation and stability, and that disruption of this structural motif perturbs myostatin signaling.

## Background

Myostatin is a member of the transforming growth factor-β (TGF-β) superfamily of growth and differentiation factors, acting as a primary negative regulator of muscle development and growth [1,2]. Myostatin over expression in animal models induces profound muscle and fat loss analogous to that seen in human cachexia syndromes [3,4]. Myostatin signaling can have negative consequences in a diseased background such as the muscular dystrophies [5] and may contribute to cachexia associated with many chronic disease states [3] including HIV [6] and cancer [7]. Hence myostatin has been suggested to hold exciting potential for inhibitory targeting in a wide range of muscle wastage diseases [8,9].

Similar to other TGF-β family members myostatin is translated as a precursor protein (MstnPP) consisting of an N-terminal signal sequence, a propeptide domain (residues 21-266) and a growth factor domain (MstnGF, residues 267-374) that contains the characteristic cystine-knot motif [10,11] and dimerises at the C-terminus via an intermolecular disulfide bond [12-14]. The propeptide region plays a chaperone role assisting folding of the growth factor region [15,16] before furin proteolysis at a conserved RSRR sequence [1,14]. The propeptide remains non-covalently associated with the mature dimer regulating its activity and targeting in the latent complex [14,17]. Myostatin remains latent until a second activating cleavage event in the propeptide region that disrupts the association [18,19].

A number of myostatin null mutations that result in a double-muscled phenotype have been documented. In one human case a child has increased muscle mass, is unusually strong and does not show any negative effects from the mutation [20]. Myostatin null mutations have also been identified in Texel sheep [1] and racing whippets [21]. Double-muscled cattle breeds such as the Belgian Blue have been recognized for almost 200 years [1]. The majority of myostatin null phenotypes result from premature stop codons and the ensuing absence of myostatin protein [22,23]. In contrast, Piedmontese cattle have the myostatin missense mutation G938A that translates to C313Y myostatin protein with the consequent loss of one of the disulphide bonds (C313-C374) involved in the characteristic TGF-β family cystine-knot structural motif. Compared to wild type, Piedmontese cattle skeletal muscle C313Y myostatin precursor protein (C313Y-MstnPP) is translated at greatly elevated levels (> 10-fold) but the C313Y mature growth factor (C313Y-MstnGF) is detected at significantly reduced levels in skeletal muscle while circulating levels are similar [10]. Refolded *E. coli *expressed C313Y-MstnGF failed to inhibit muscle cell proliferation and acted as a dominant negative inhibitor of wild type (WT) myostatin [10].

**Figure 1:**
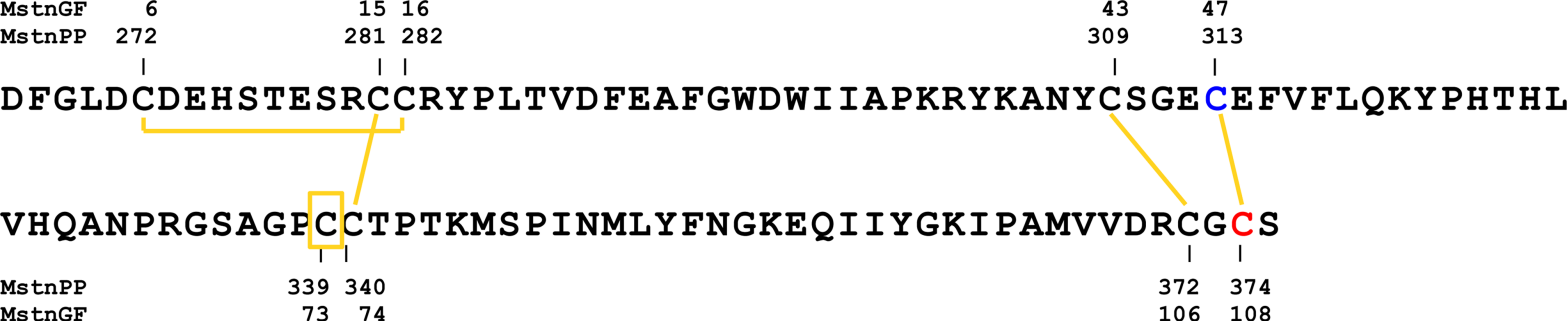
**The myostatin growth factor primary structure**. Disulphide bonds are shown in yellow; C313 is highlighted in blue, C374 in red and the dimerisation C339 is boxed. Sequence numbering for the full-length precursor myostatin (MstnPP) and processed myostatin growth factor alone (MstnGF) are shown. MstnPP numbering is used throughout this paper.

The MstnGF monomer contains four disulphide bonds (Figure 1, yellow), three of which are involved in an intricate cystine-knot motif (C281-C340, C309-C372 and C313-C374); C339 forms the intermolecular dimerisation disulphide bond. We have investigated the *in vitro *structure and stability of C313Y-MstnPP and additional disulphide mutant proteins C313A-MstnPP and C374A-MstnPP. C313Y recapitulates the mutation found in Piedmontese myostatin, C313A disrupts the C313-C374 disulphide bond without introducing a large tyrosine residue minimizing steric interference and C374A removes the same disulphide bond through mutation of the partner cysteine residue. The results presented here show a significant decrease in C313Y myostatin covalent disulphide linked dimerisation, lowered thermal stability and a failure to inhibit myoblast proliferation relative to WT. This research indicates that myostatin structural stability and covalent dimerisation are maintained by the cystine-knot and suggests that this structural motif is required for receptor-mediated signaling by the myostatin growth factor *in vivo*, with its absence leading to a double-muscled phenotype.

## Results

### C313 mutations decrease myostatin disulphide-linked covalent dimerisation

**Figure 2:**
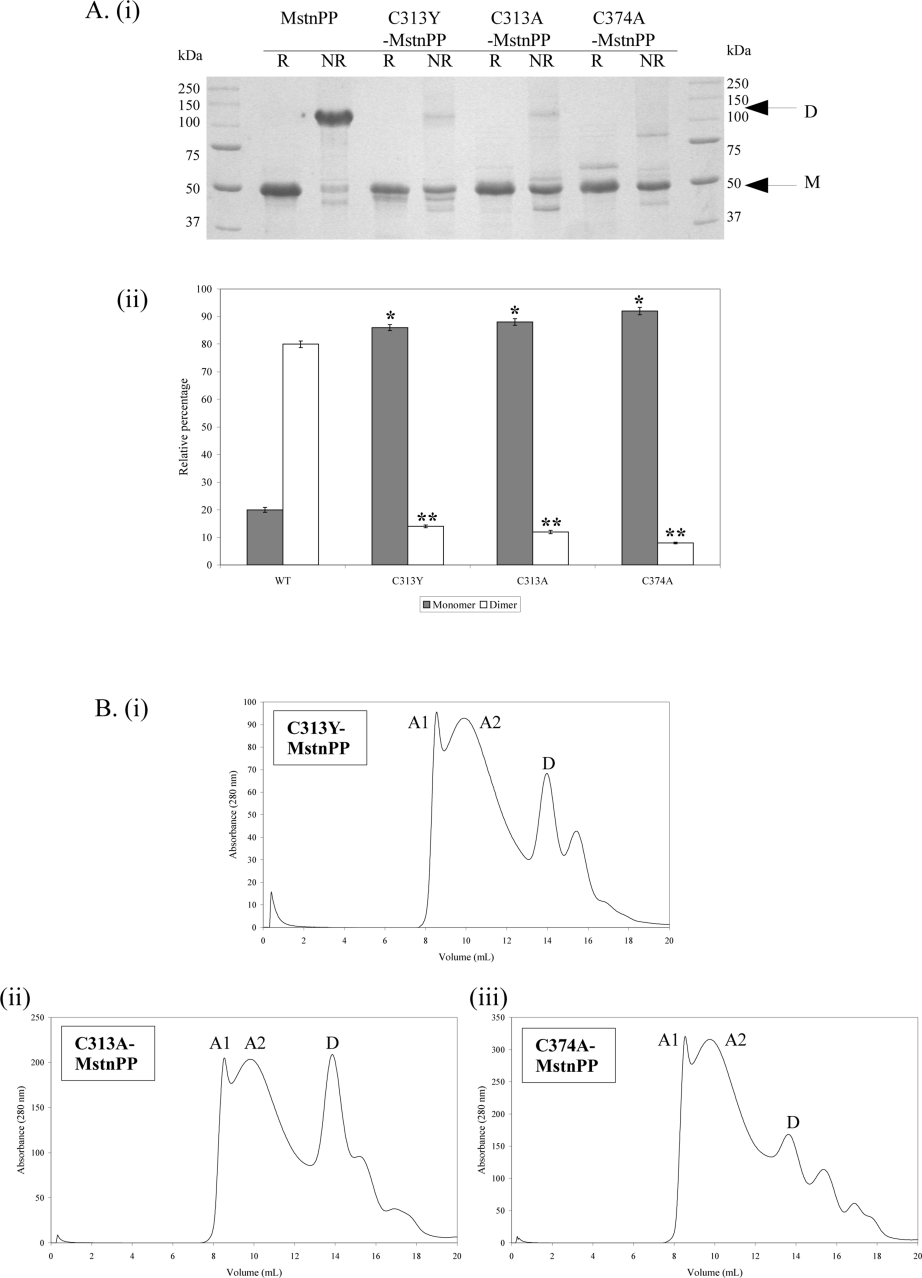
**Mutations in the myostatin precursor protein inhibit covalent dimerisation and increase aggregation during refolding**. A. (i) Reducing (R) versus non-reducing (NR) SDS-PAGE (12%) analysis of dimer (D) and monomer (M) ratios for purified precursor protein dimers after gel filtration chromatography; (ii) Quantitative densitometry analysis of relative proportions of monomer and dimer for each protein. Percentages are calculated relative to the fully reduced monomer. Statistical significance was calculated using a paired Student's t-test where * is P < 0.0001 for monomers relative to the WT monomer and **<0.0001 for dimers relative to the WT dimer. B. Gel filtration chromatography of (i) C313Y-MstnPP; (i) C313A-MstnPP and (iii) C374A-MstnPP. Peaks are labeled as follows: A1, void volume aggregates; A2, lower molecular weight aggregates; D, dimer.

All C313 mutant myostatin proteins could be refolded and purified using the WT protocol of heparin affinity chromatography followed by gel filtration chromatography [24]. Disulphide-linked dimerisation is significantly decreased in the mutant proteins, as seen by SDS-PAGE densitometric analysis (Figure 2A). A proportion of the C313 myostatin mutants eluted as dimer during gel filtration chromatography (Figure 2B, peak D) but they are not disulphide linked, owing to their monomeric Mw displayed on non-reducing SDS-PAGE. In addition, gel filtration chromatograms highlight increased aggregation during refolding for all mutant proteins (Figure 2B, peaks A1 and A2) compared to WT [24].

### C313 mutant myostatin precursor protein structures are similar to WT but show decreased stability

**Figure 3:**
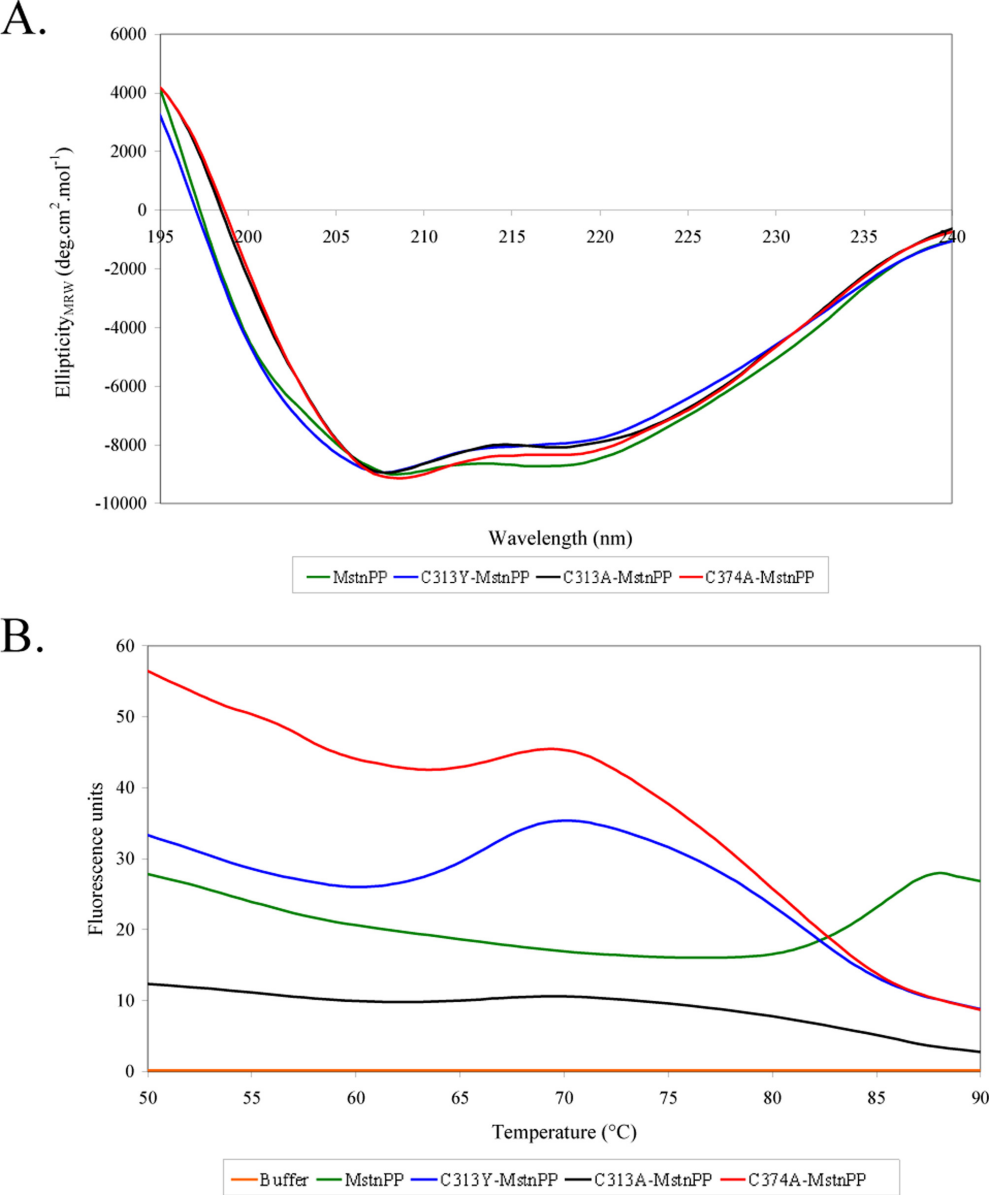
**Myostatin precursor protein disulphide mutants are structurally similar to the WT but have decreased thermal stability**. A. Circular dichroism spectra from 195 to 240 nm of MstnPP (green), C313Y-MstnPP (blue), C313A-MstnPP (black) and C374A-MstnPP (red). B. Fluorescence-based thermal shift assay; MstnPP (green); C313Y-MstnPP (blue); C313A-MstnPP (black); C374A-MstnPP (red); and buffer only control (orange).

The circular dichroism (CD) spectra of C313Y-MstnPP, C313A-MstnPP and C374A-MstnPP are broadly comparable to that of MstnPP (Figure 3A), but show decreased absorption at 217 nm indicating that disruption of the C313-C374 disulphide bond by mutation of C313 or C374 compromises the integrity of β-sheet structures. The overall similarity of the MstnPP C313 mutants' CD spectra to WT and other TGF-β family propeptide and precursor protein spectra [25,26] indicates refolding was successful. Sypro Orange fluorescence thermal shift assays [27] show that C313Y-, C313A- and C374A-MstnPP all have reduced thermal stability with major unfolding transitions approximately 20°C lower than the 86°C of MstnPP, at 66, 67 and 67°C respectively (Figure 3B).

### Structural modeling and tryptic digestion of C313Y-myostatin precursor protein suggest structural perturbation

**Figure 4:**
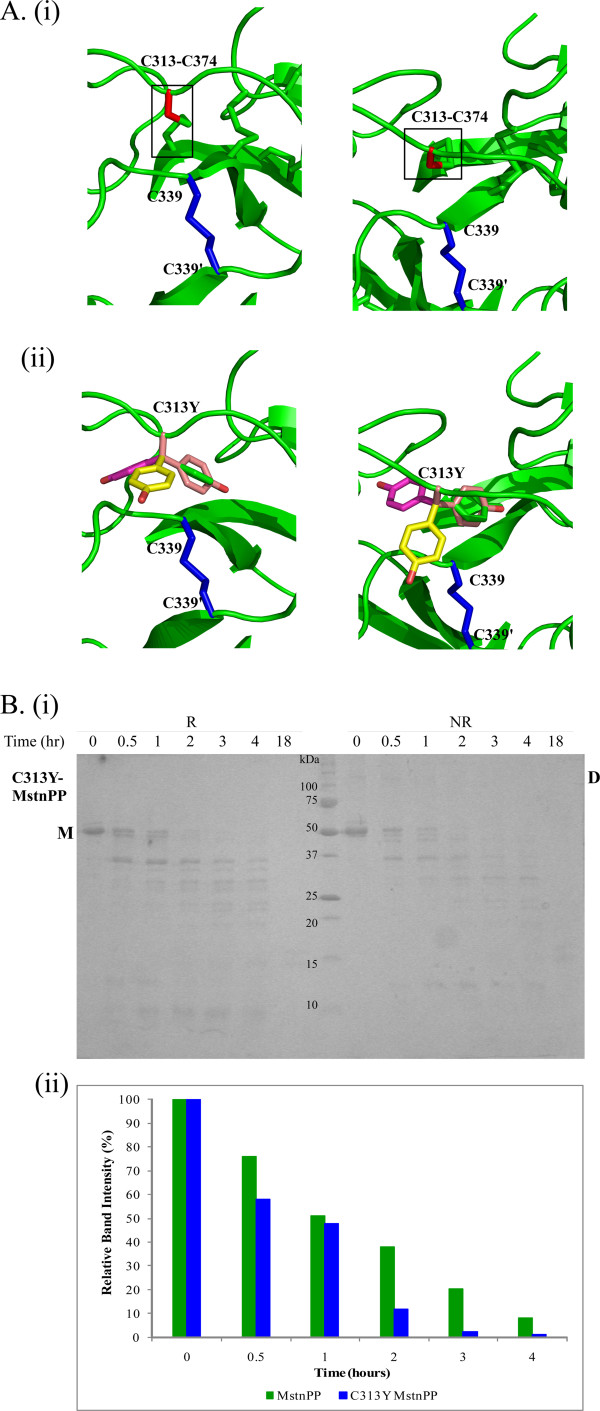
**Structural modeling and tryptic digestion of C313Y-MstnPP suggest structural perturbation**. A. Orthogonal structural representations of the environment of residue 313 within: (i) The MstnGF crystal structure (green ribbons) highlighting the native C313-C374 disulphide bond (boxed, C313 red; C374 green stick representation) (ii) The modeled C313Y-MstnGF structure with C313 replaced by Tyr showing a superposition of the Tyr sidechain favoured rotamers (Chi 1 angles -60°, 180°, 60°). The intermolecular disulphide (C339-C339') is shown in blue stick representation. The right-hand view is rotated ~90° around the X-axis relative to the left-hand view. B. (i) Limited tryptic proteolysis of C313Y-MstnPP. Reducing (R) and non-reducing (NR) SDS-PAGE showing bands as indicated: M, monomer; D, dimer. Tryptic digestion was performed at 37°C with a myostatin:trypsin ratio of 100:1(w/w) and samples were taken at 0, 0.5, 1, 2, 3, 4 and 18 hour time points. (ii) Quantification of trypsin proteolysis of C313Y-MstnPP and MstnPP [24], as a function of time, normalised to the concentration at time zero.

In addition to disruption of the cystine-knot motif the C313Y substitution will perturb myostatin structure. Modeling the C313Y-MstnGF structure by directly substituting a tyrosine sidechain for cysteine at position 313 in the MstnGF crystal structure [11] using Coot [28] showed that all energetically favoured tyrosine side chain rotamers cannot occur without significant structural rearrangement owing to steric interference with nearby regions of the structure (Figure 4A). The location of 313 in combination with disruption of the cystine knot suggests an increase in structural flexibility on removal of disulphide bond induced structural constraints. The resistance of C313Y-MstnPP to limited proteolysis was investigated by incubation with trypsin at 37°C followed by analysis using reducing (R) and non-reducing (NR) SDS-PAGE. The C313Y-MstnPP tryptic product pattern is very similar to WT indicative of a similar overall fold [24]. However, the rate of C313Y-MstnPP proteolysis is increased relative to MstnPP consistent with improved protease accessibility owing to decreased structural compactness and/or increased structural flexibility in combination with decreased dimerisation (Figure 4B). The C313Y substitution disrupts the cystine-knot motif leading to improved protease accessibility. The MstnGF proteolytic product is not observed as trypsin fails to cleave at the RSRR furin site (unpublished results).

### Furin processing and secondary structure of C313Y-myostatin are maintained

**Figure 5:**
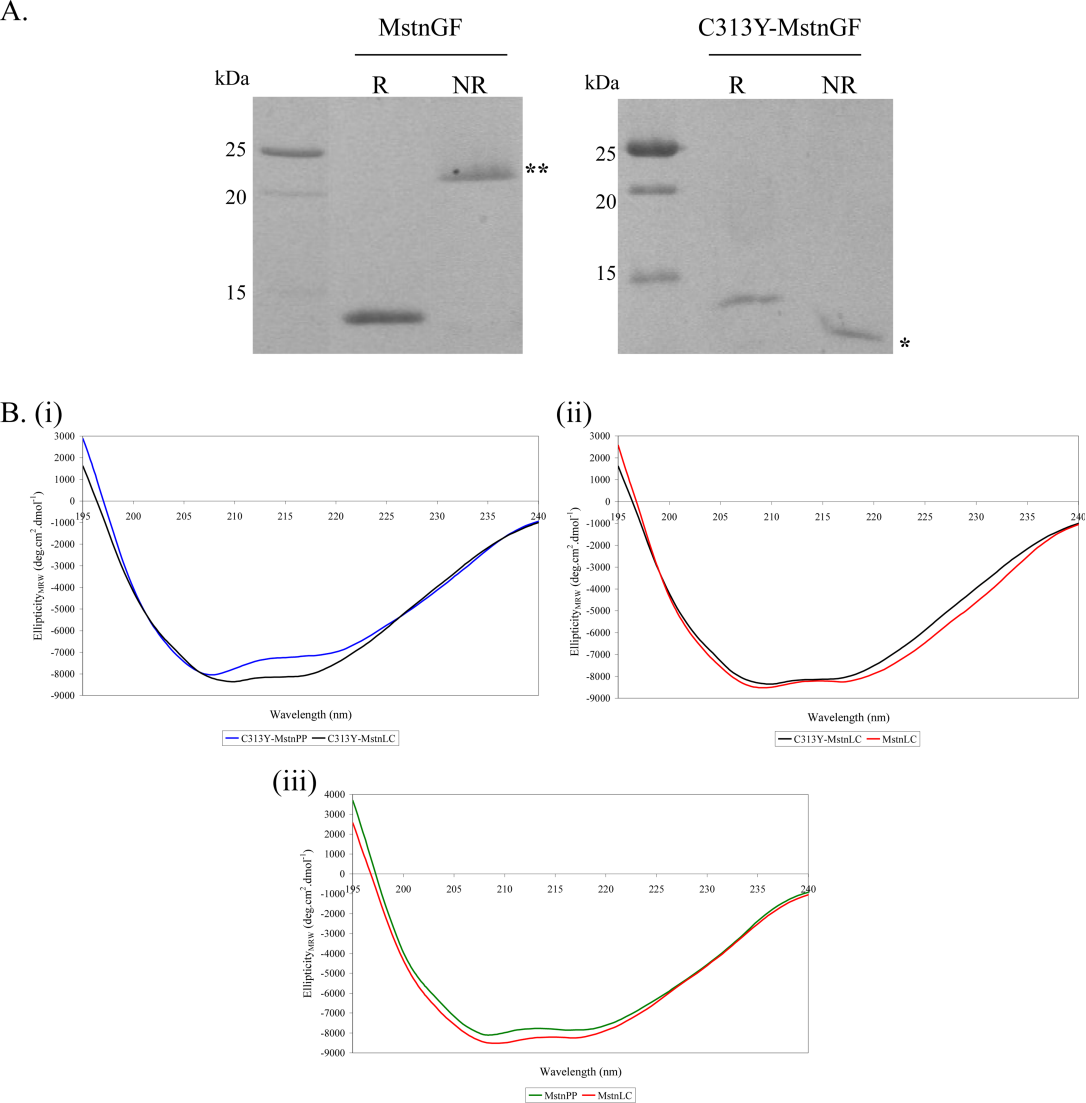
**Furin proteolytic processing of C313Y-MstnPP and C313Y-MstnGF secondary structure is maintained**. A. Analysis of (i) WT and (ii) C313Y precursor protein furin cleavage products by reducing (R) and non-reducing (NR) 15% SDS-PAGE. WT growth factor dimer (**) and C313Y growth factor monomer (*) under non-reducing conditions are indicated. B. Circular dichroism analysis of MstnLC and C313Y-MstnLC from 195 to 240 nm. (i) Comparison of C313Y-MstnPP (blue) and C313Y-MstnLC (black). (ii) Comparison of WT MstnPP (green) and MstnLC (red). (iii) Comparison of C313Y-MstnLC (black) and MstnLC (red).

**Figure 6:**
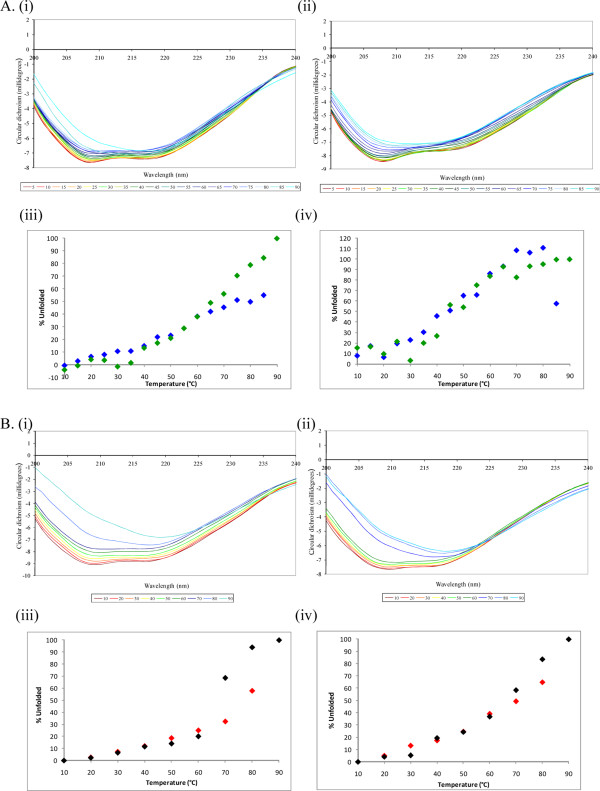
**Thermal unfolding of WT and C313Y myostatin followed by CD suggests similar behavior at high temperatures and decreased latent complex stability**. A. Myostatin precursor protein circular dichroism thermal unfolding spectra (i) MstnPP and (ii) C313Y-MstnPP. Normalised unfolding analysis for MstnPP (blue) and C313Y-MstnPP (green) at (iii) 209 nm and (iv) 217 nm. B. Myostatin latent complex circular dichroism thermal unfolding spectra (i) MstnLC and (ii) C313Y-MstnLC. Normalised unfolding analysis for MstnLC (red) and C313Y-MstnLC (black) at (iii) 209 nm and (iv) 217 nm.

*In vivo*, MstnPP is processed to give the propeptide/growth factor dimer latent complex for export. *In vitro *incubation of MstnPP and C313Y-MstnPP with furin resulted in proteolytic processing of the proprotein to the mature growth factor for both WT and C313Y myostatin as evident by the monomeric growth factor product band at 12 kDa under reducing conditions. As expected MstnGF is a disulphide-linked dimer of 25 kDa under non-reducing SDS-PAGE conditions but the C313Y growth factor fails to form a disulphide-linked dimer and exhibits increased mobility on SDS-PAGE (Figure 5A). The failure of C313Y-MstnGF to covalently dimerise is consistent with results observed for C313Y-MstnPP (Figure 1A). CD analysis shows that the furin processing of C313Y-MstnPP to C313Y latent complex (C313Y-MstnLC) increases the proportion of β-sheet secondary structure (Figure 5B (i)) inferring some structural rearrangement upon proteolytic processing. This β-sheet increase is a reversal of the decrease seen for C313Y-MstnPP compared to MstnPP (Figure 3A). The secondary structure of C313Y-MstnLC is more similar to WT MstnLC than to C313Y-MstnPP from which it was processed (Figure 5B (ii)). In contrast the spectra of WT MstnPP and MstnLC are largely similar (Figure 5B (iii)). Comparison of CD thermal melting curves for C313Y-MstnPP and MstnPP showed no distinctive differences in unfolding behaviour except at high temperature (Figure 6A). The CD melting curves are representative of a complex (i.e. not two-state, or single intermediate) unfolding mechanism [29], as expected for myostatin that aggregates and forms amyloid at elevated temperatures [24]. CD thermal unfolding of WT and C313Y-MstnLC suggests that the mutant latent complex has decreased stability compared to the WT (Figure 6B) though the differences are not as large as observed in Sypro Orange assays that reports on hydrophobic core solvent accessibility (Figure 3B).

### C313Y myostatin growth factor proteolytically processed from C313Y-MstnPP fails to inhibit myoblast proliferation

**Figure 7:**
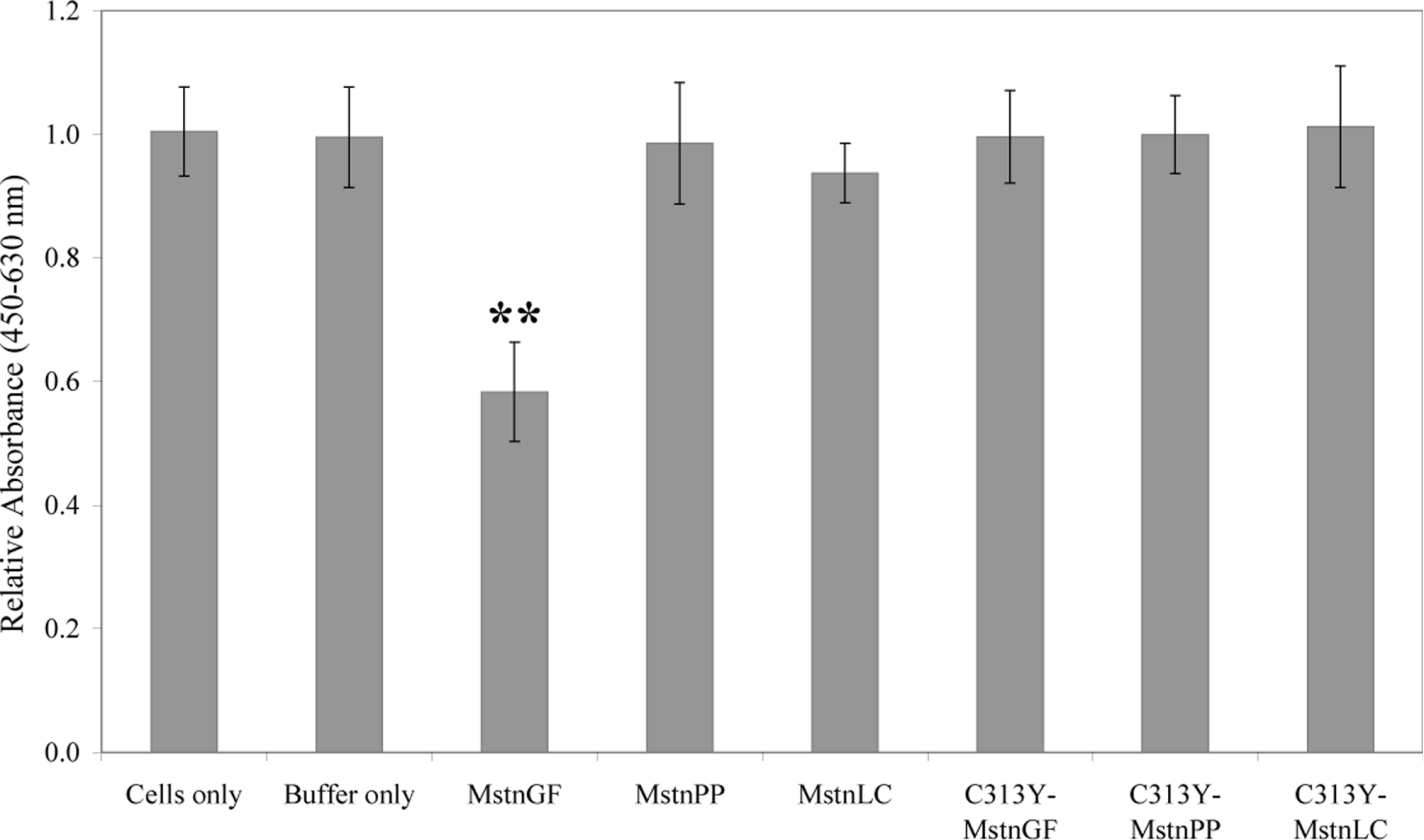
**C313Y-MstnGF is unable to inhibit the proliferation of C2C12 myoblasts**. The difference in absorbance at 450 and 630 nm correlates to cell density after incubation with WT or C313Y myostatin growth factor, precursor protein or latent complex. Cells incubated in media in the absence of protein but with buffer (Buffer only) or without buffer (Cells only) were used as controls. Error bars represent the standard error of the mean for triplicate samples from three independent experiments for WT samples and two independent experiments for C313Y. Statistical significance was calculated using a paired Student's t-test where ** P < 0.05.

C313Y myostatin activity was measured by C2C12 myoblast proliferation assays [30-32]. Growth factor was obtained by cleavage of MstnPP and C313Y-MstnPP with furin convertase and acid-induced dissociation [19] of the propeptide-growth factor latent complex. C313Y-MstnGF, C313Y-MstnPP and C313Y-MstnLC do not inhibit myoblast proliferation (Figure 7). These observations are consistent with studies of bovine C313Y myostatin growth factor also expressed in *E. coli *but refolded directly as the mature growth factor protein [10]. Positive control MstnGF inhibits C2C12 myoblast proliferation compared to cells only, buffer only, MstnPP and MstnLC controls (Figure 7). Wild type inhibition was less dramatic than that seen in assays performed previously [10] owing to incomplete acid-induced dissociation of the propeptide in the latent complex [19].

## Discussion

**Figure 8:**
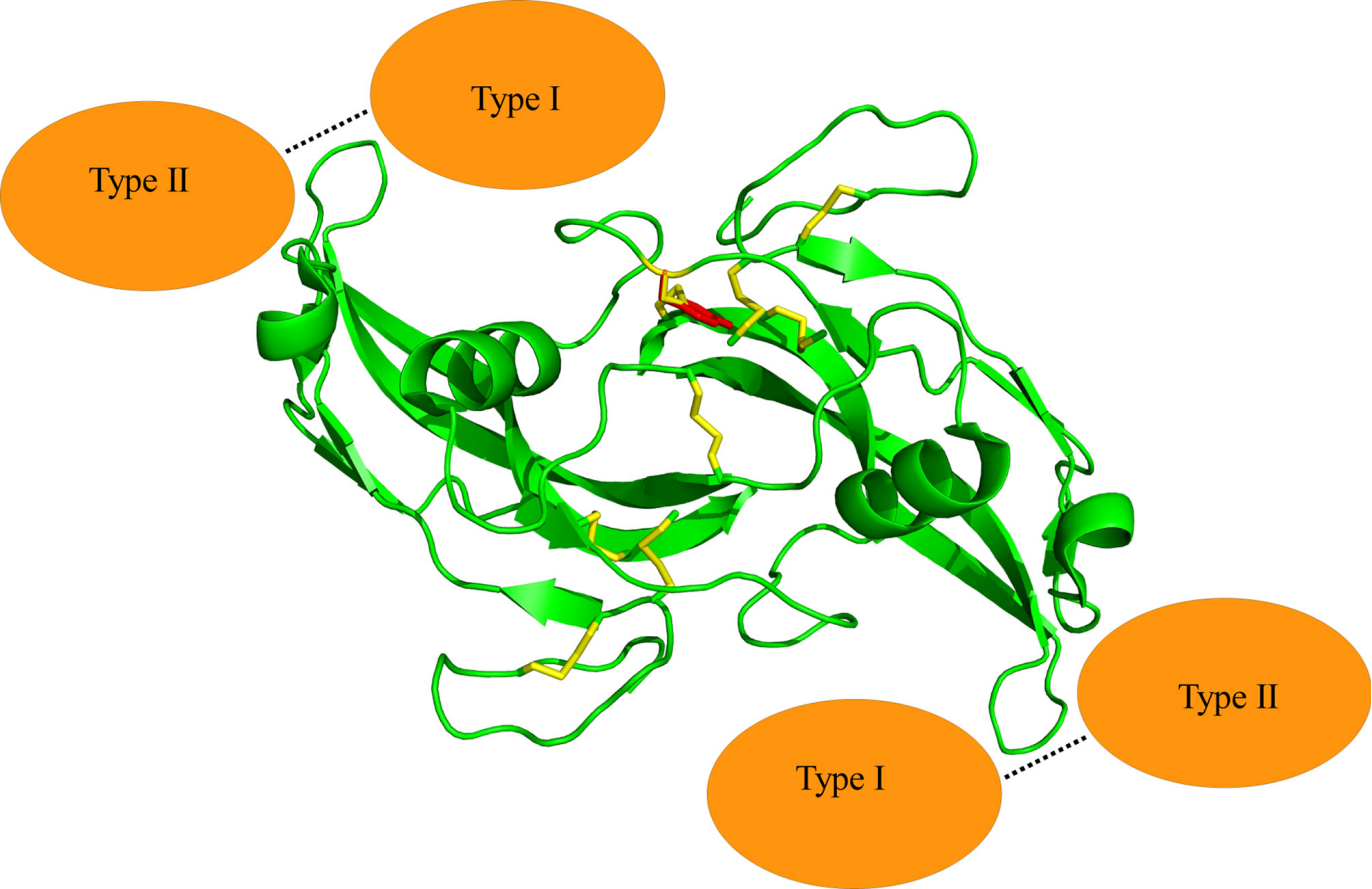
**The MstnGF structure showing the proximity of the C313Y mutation site to the putative Type I receptor binding site**. The C313Y site is shown in one monomer only for clarity. Disulphide bonds are shown in yellow and the modeled C313Y in red. Receptor binding is modeled by homology with interactions documented for the TGF-β growth factors [11].

Myostatin contains the canonical cystine-knot motif and dimerises via an intermolecular disulphide bond as found for nearly all TGF-β family members. Even though the overall structures of the myostatin C313 mutants are broadly similar to WT there is significantly less covalently linked dimeric C313 mutant myostatin. Gel filtration chromatography shows that C313 mutant myostatin dimerisation can occur, but it is not mediated by an intermolecular disulphide bond but occurs, presumably, via interactions at the hydrophobic dimerisation interface [11]. The C313 mutation site is structurally adjacent to the inter-molecular disulphide bond and increased structural flexibility or rearrangement in the absence of the C313-C374 disulphide will lead to structural distortion in the growth factor domain affecting disulphide-linked dimerisation. This structural distortion may interfere with interactions between the myostatin growth factor domain and the propeptide domain and/or with its receptor. This analysis with purified recombinant myostatin is consistent with results showing reduced levels of dimeric myostatin in Piedmontese cattle skeletal muscle compared to WT controls even in the context of a greatly elevated mutant precursor myostatin concentration [10]. The chaperone-like role of the myostatin propeptide region for correct folding of the growth factor motif [15,16] means that recombinant C313Y-MstnGF prepared from furin cleavage of C313Y-MstnPP more closely mimics *in vivo *production of C313Y-MstnGF than refolding of recombinant growth factor domain expressed in isolation. The absence of signaling activity of C313Y-MstnGF, derived from proteolytically processed C313Y-MstnPP, causing no effect on C2C12 myoblast proliferation, confirms the importance of the cystine knot motif for myostatin function. All the disulphide mutants, C313Y-MstnPP, C313A-MstnPP and C374A-MstnPP, exhibit reduced thermal stability and have an increased propensity to aggregate. These results are consistent with the cystine-knot being required for the high stability of myostatin as observed for other members of this growth factor super family [33,34]. Removal of the myostatin C313-C374 disulphide leads to an increase in susceptibility of C313Y myostatin to proteolysis, through decreased β-sheet structure and presumed increased flexibility. Examination of the structural environment around residue 313 reveals that perturbations in the WT myostatin structure are required to accommodate the larger C313Y tyrosine. Analysis of C313A and C374A mutations confirms that it is disruption of the cystine-knot by removal of the C13-C374 disulphide bond and not by the introduction of the tyrosine that is the dominant factor in the decreased stability and reduced disulphide-linked dimerisation of C313Y myostatin. Myostatin elicits cell signaling via type I and type II Ser/Thr kinase transmembrane receptors [1]. C313 is localized near the concave surface of myostatin, close to the type I receptor binding site [35] (Figure 8). It is not possible to exactly predict the structural consequences of the C313Y mutation; disruption of the cystine-knot and decreased covalent dimerisation likely results in a less compact structure that is then unsuitable for receptor activation. Alternative possible mechanisms for the loss of C313Y myostatin function are that structural alterations owing to the C313Y mutation result in increased binding to myostatin negative regulators such as follistatin or decorin [14,36], or altered targeting [17].

## Conclusion

In summary, *in vitro *characterization of C313Y myostatin shows that removal of the C313-C374 disulphide bond by site-directed mutagenesis decreases myostatin covalent dimerisation with subsequent loss of activity and lowered stability. An intact cystine-knot structural motif is essential for myostatin dimerisation and function.

## Methods

### Production of C313Y, C313A and C374A myostatin proteins

Mutant MstnPP expression constructs were prepared by PCR from the WT myostatin construct described previously [24]. PCR fragments were cloned into a modified pET vector via *BamHI *and *XhoI *restriction sites and constructs confirmed by sequencing. The *E. coli *BL21 (DE3) expression, refolding and purification of the mutant proteins was conducted using the procedure previously described for WT myostatin [24].

### Analysis of disulphide-linked dimerisation by reducing and non-reducing SDS-PAGE

Intermolecular disulphide formation was analysed by visualising purified myostatin (1 mg/ml) on reducing (R) versus non-reducing (NR) SDS-PAGE. All conditions were identical except for the presence (R) or absence (NR) of reducing agent (β-mercaptoethanol, 1.4 M) in the SDS sample buffer. Band densities were quantified with a correction for background staining using gel densitometry. The Student's t-test was performed using GraphPad Prism (GraphPad Software, Inc) using triplicate gels.

### Circular dichroism spectroscopy and thermal denaturation

CD spectra in the far-UV region (195-240 nm) were measured for myostatin (1 mg/mL) with a Chirascan CD spectrometer (Applied Biophysics) using a 0.1 mm cell at 4°C. For each sample 20 spectra of 1 nm interval were collected every 2.5 seconds, followed by baseline subtraction, averaging and smoothing. For CD thermal denaturation, 1 nm/2.5 second readings were taken at every 5°C (precursor protein) or 10°C (latent complex) increase in temperature from 5 - 90°C with a 30 second equilibration time at each temperature and a tolerance level of 0.2°C. The change in C313Y-MstnPP CD signal was normalized by the decreased initial proportion of β-sheet for, relative to MstnPP, before thermally-induced denaturation (Figure 3A).

### Fluorescence-based thermal shift assays

Sypro Orange (Sigma) was diluted 100x according to manufacturer's instructions in milliQ H_2_O with 2 μL then added to 18 μL myostatin (1 mg/ml in 50 mM Tris-HCl, 150 mM NaCl, pH 8.5). Negative controls contained buffer and dye only. A Rotor-Gene 6000 thermocycler (Corbett) was used for analysis with excitation at 470 nm and fluorescence emission measured at 555 nm over increasing temperature from 30 to 95°C in 1°C increments. Melting temperatures were calculated with the Rotor-Gene 6000 software.

### Structural modeling

The myostatin coordinates from the myostatin/follistatin complex structure (PDB code: 3HH2) were used to model the C313Y, C313A and C374A mutations with the amino acid substitution and rotamer tools in Coot [28]. Structural figures were prepared with PyMol [37].

### Protease resistance analysis

Protease resistance was assayed by incubating C313Y-MstnPP at a w/w ratio of 100:1 trypsin (bovine pancreas, Sigma) at either 4°C, room temperature or 37°C. Samples were taken after 0.5, 1, 2, 3, 4 and 18 hours (overnight), denatured, and then analysed by reducing and non-reducing SDS-PAGE. The decrease in concentration of full-length C313Y-MstnPP compared to MstnPP [24] as a function of time, normalised to the concentration at time zero, was quantified by gel densitometry using Image-J software [38] Owing to the different relative proportions of MstnPP and C313Y-MstnPP monomer and dimer under non-reducing conditions, the reduced SDS-PAGE monomer bands were quantified enabling direct comparison between the two proteins.

### Furin cleavage

Purified myostatin precursor protein dimer, in HEPES pH 7.5, 150 mM NaCl buffer, was concentrated to 10 mg/mL and furin cleavage buffer (50 mM HEPES pH7.5 and 1 mM CaCl_2_) was added to a final volume of 250 μL per 1 mg of protein. Human furin convertase (Sigma, 2 U/μL) was added at a ratio of 1 μL furin to 100 μg protein. The reaction was incubated at 30°C for 64 hours and subsequently centrifuged at 17,000 × g, 4°C for 5 minutes to remove precipitated protein.

### C2C12 myoblast proliferation myostatin activity assay

C2C12 mouse myoblasts were cultured in Advanced Dulbecco's Modified Eagle's Medium (4.5 g/L D-glucose and 110 mg/L sodium pyruvate; Gibco, Invitrogen) supplemented with 10% foetal calf serum, 4 mM *L*-glutamine and penicillin/streptomycin. Cells were incubated at 37°C in a 5% CO_2 _humidified environment and passaged at 80-90% confluency. Cells were plated in fresh medium in optical bottom 96-well plates at a density of 1,000 cells/well. After 24 hours, media was removed and 100 μL/well fresh media containing myostatin (10 μg/mL) was added. Equivalent concentrations of the untreated furin digest and MstnPP were used. Activation of the MstnGF was performed by acid treatment as described previously [19]. Cells were incubated with protein for 72 hours and cell growth was measured using the WST-1 Cell Proliferation Assay (Roche). WST-1 (10 μL) was added to each well and incubated for a further 3 hours. Absorbance at 450 nm and 630 nm was measured in a PowerWave XS 96-well plate reader (BioTek Instruments, Inc). Cells incubated in media only and media containing furin cleavage buffer only were used as negative controls. Three independent experiments each with triplicate wells were used for each condition for WT; two independent experiments were conducted in triplicate for C313Y. The Student's t-test was performed using GraphPad Prism (v5.04, GraphPad Software, San Diego, California, USA).

## Competing interests

The authors declare that they have no competing interests.

## Authors' contributions

CSS and AJSS conceived of the study, participated in its design and coordination, interpreted the data and wrote the manuscript. CSS conducted the experimental work. Both authors have read and approved the final manuscript.
